# Supplementary material for: Immunomics-guided biomarker discovery for human liver fluke infection and infection-associated cholangiocarcinoma
Source: Nat Commun. 2025 Jul 1;16:5965. doi: 10.1038/s41467-025-61043-2 (PMC12218120; doi:10.1038/s41467-025-61043-2)
Supplement: Supplementary file 1 — Supplementary Information [file 41467_2025_61043_MOESM1_ESM.pdf]

Supplementary Figures and tables for:

Immunomics-guided biomarker discovery for human liver fluke infection and infection-associated cholangiocarcinoma

Lakkhana Sadaow, PhD[1], Rutchanee Rodpai, PhD[1], Michael J Smout, PhD[2], Rie Nakajima, MSc[3], Patcharaporn Boonroumkaew, PhD[1], Javier Sotillo, PhD[4], Bemnet A Tedla, PhD[2], Vor Luvira, MD[5], Amnat Kitkhuandee, MD[5], Krisada Paonariang, MD[5], Wattana Sukeepaisarnjaroen, MD[6], Hiroshi Yamasaki, PhD[7], Sutas Suttiprapa, PhD[8], Thewarach Laha, PhD[1], Banchob Sripa, PhD[8], Rafael de Assis, PhD[3], Aarti Jain, MSc[3], Wannaporn Ittiprasert, PhD[9], Victoria H. Mann, PhD[9], Yide Wong, PhD[2], Philip L Felgner, PhD[3], Wanchai Maleewong, PhD[1], Paul J Brindley, PhD[9*], Alex Loukas, PhD[2*], Pewpan M. Intapan, MD[1]

[1]Department of Parasitology, Faculty of Medicine, and Mekong Health Science Research Institute, Khon Kaen University, Khon Kaen, Thailand.
[2]Australian Institute of Tropical Health and Medicine, James Cook University, Cairns, QLD, Australia
[3]Vaccine R&D Center, Department of Physiology and Biophysics, University of California Irvine, Irvine, CA, USA
[4]Parasitology Reference and Research Laboratory, Centro Nacional de Microbiologia, Instituto de Salud Carlos III, Majadahonda, Madrid, Spain
[5]Department of Surgery, Faculty of Medicine, Khon Kaen University, Khon Kaen, Thailand
[6]Department of Medicine, Faculty of Medicine, Khon Kaen University, Khon Kaen, Thailand
[7]Department of Parasitology, National Institute of Infectious Diseases, Tokyo, Japan
[8]Tropical Disease Research Center, Department of Tropical Medicine, Faculty of Medicine, Khon Kaen University, Khon Kaen, Thailand
[9]Department of Microbiology, Immunology and Tropical Medicine, School of Medicine and Health Sciences, George Washington University, Washington, DC, USA

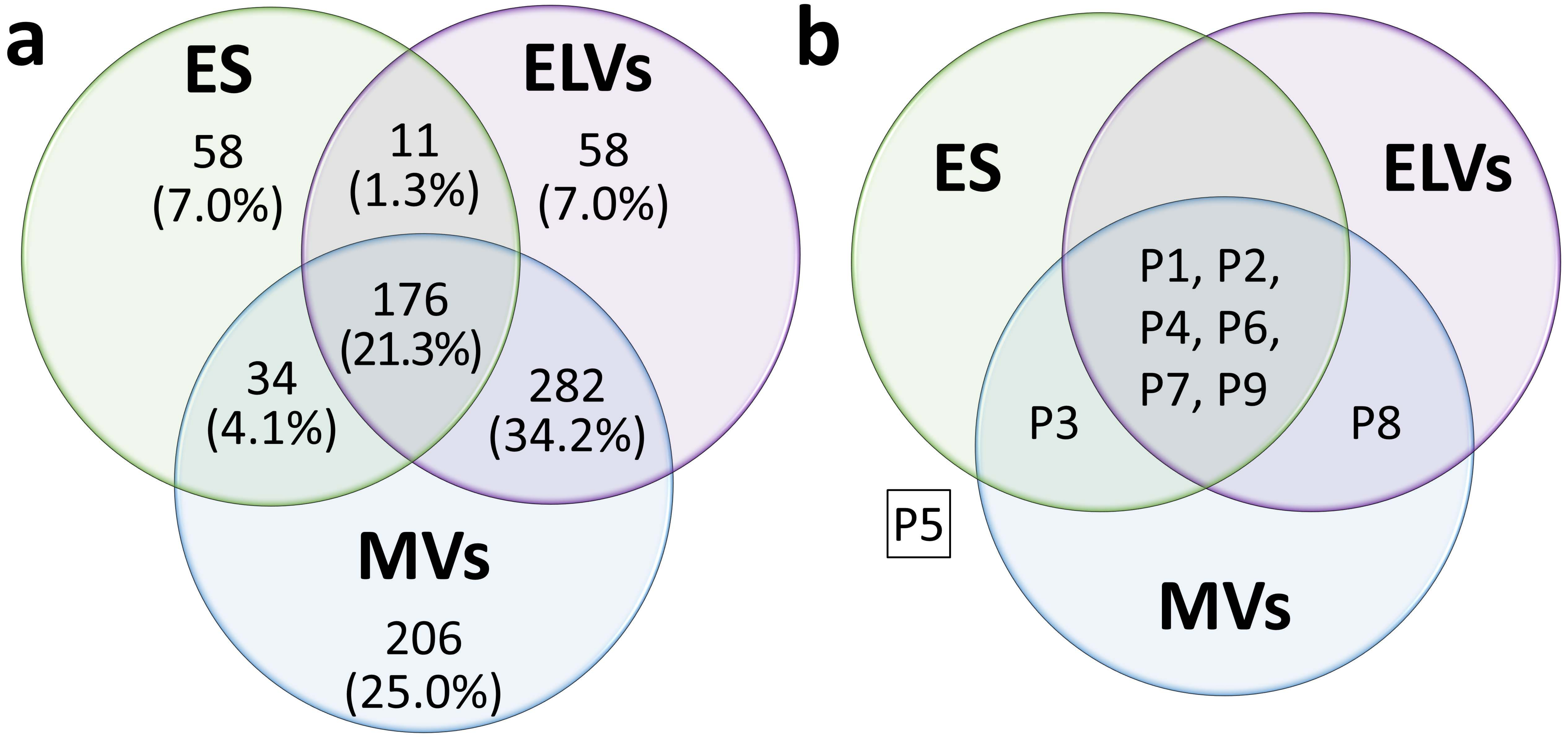

Supplementary Figure 1. Venn diagrams of Secreted proteomes of *O. viverrini*. Proteins that were shared between *Opisthorchis viverrini* excretory/secretory (ES) products, exosome-like vesicles (ELVs), and microvesicles (MVs). Total proteins identified using tandem mass spectrometry (a) and proteins P1-9 selected for further investigation (b). P5 is a fluke gut mucinase protein and not detected in these secreted protein groups.

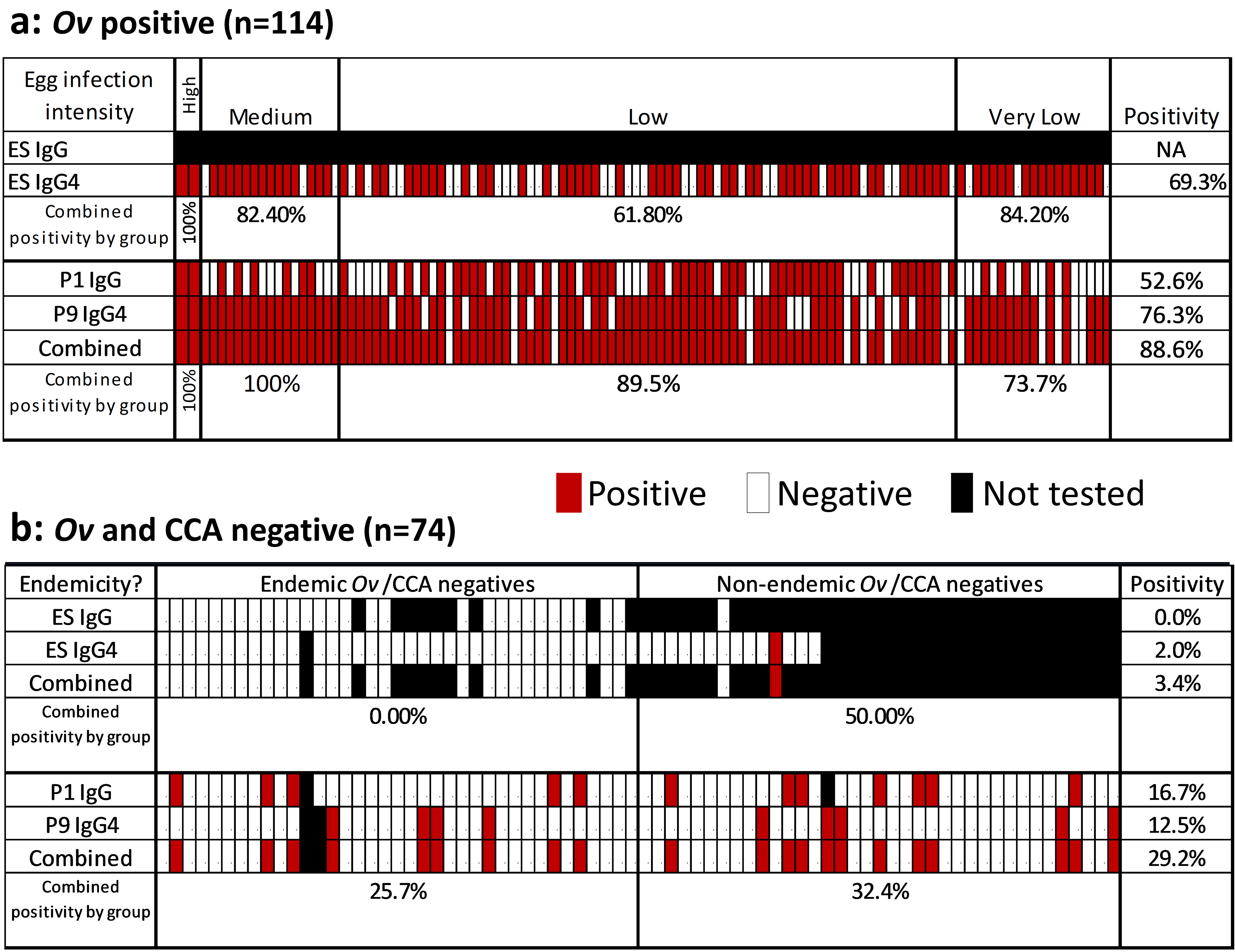

Supplementary Figure 2. Comparison of P1-IgG, P9-IgG4 and ES-IgG4 immunochromatographic tests (ICTs) for diagnosing *Opisthorchis viverrini* infections. Heatmaps are representative of the data presented in figure 4 and show positive ICT signals above the cutoff in red, negative signals below the cutoff in white, and not tested in black. *O. viverrini* positive samples (a); *O. viverrini* and cholangiocarcinoma (CCA) negative samples (b).

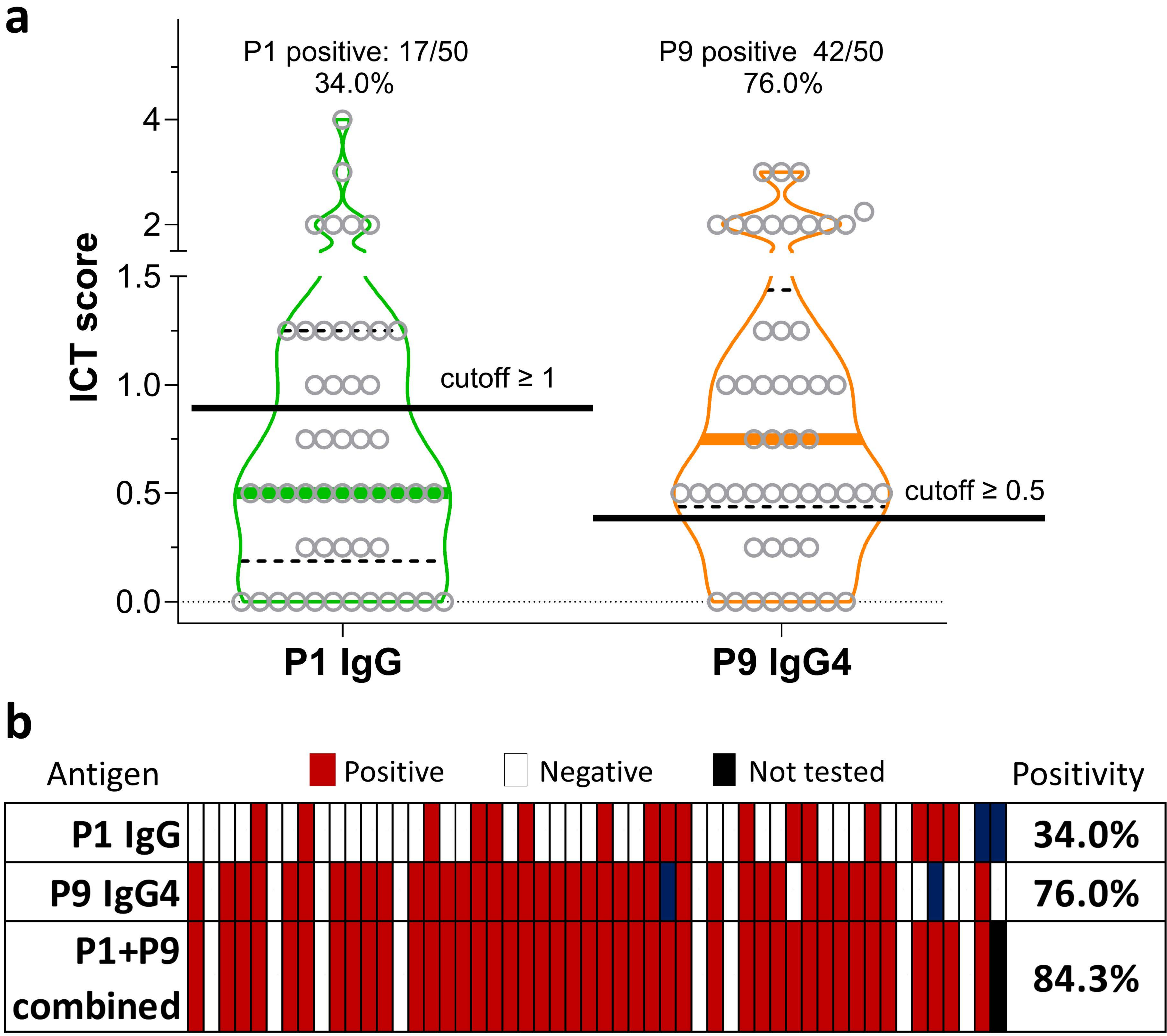

Supplementary Figure 3. Comparison of P1-IgG and P9-IgG4 immunochromatographic tests (ICTs) for diagnosing *Opisthorchis viverrini* infections in a second cohort of O. viverrini infected subjects who had a positive diagnosis of eggs in the feces but egg counts were not quantified and only recorded as FECT-positive or negative.
ICT score plotted as violin graph with individual data points as grey circles (a). The median values are denoted by thick colored lines and quartiles as black dashed lines. Cutoff marked with thick black line. Heatmaps (b) show positive ICT signals above the cutoff in red, negative signals below the cutoff in white, and not tested in black

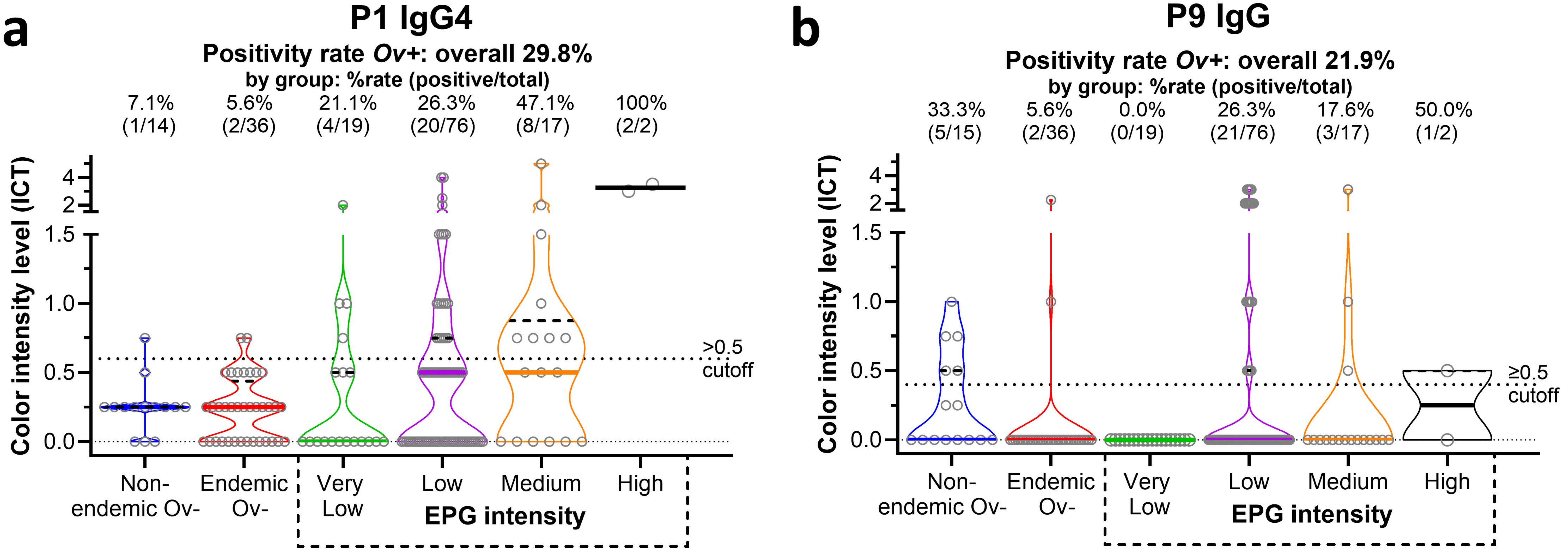

Supplementary Figure 4. Association between *Opisthorchis viverrini* infection intensity and positivity rate of point-of-care immunochromatography tests (PoC-ICTs) for P1 and P9 antigens. P1-IgG4 (a) and P9-IgG (b) ICT reactivity scores of 114 *O. viverrini* infected subjects ranked by intensity of eggs per gram of faeces (EPG) and endemic (Thailand) and non-endemic (Australia + Thailand) uninfected controls. Uninfected control totals vary as not all subjects were tested due to sample limitations. The median values are denoted by thick colored lines and quartiles as black dashed lines. EPG intensity was categorized as follows: Very low, 0-100 EPG; Low, 100-1000 EPG; Medium, 1,000-10,000 EPG; High, >10,000 EPG. Positivity cut-off is marked with a dotted line.

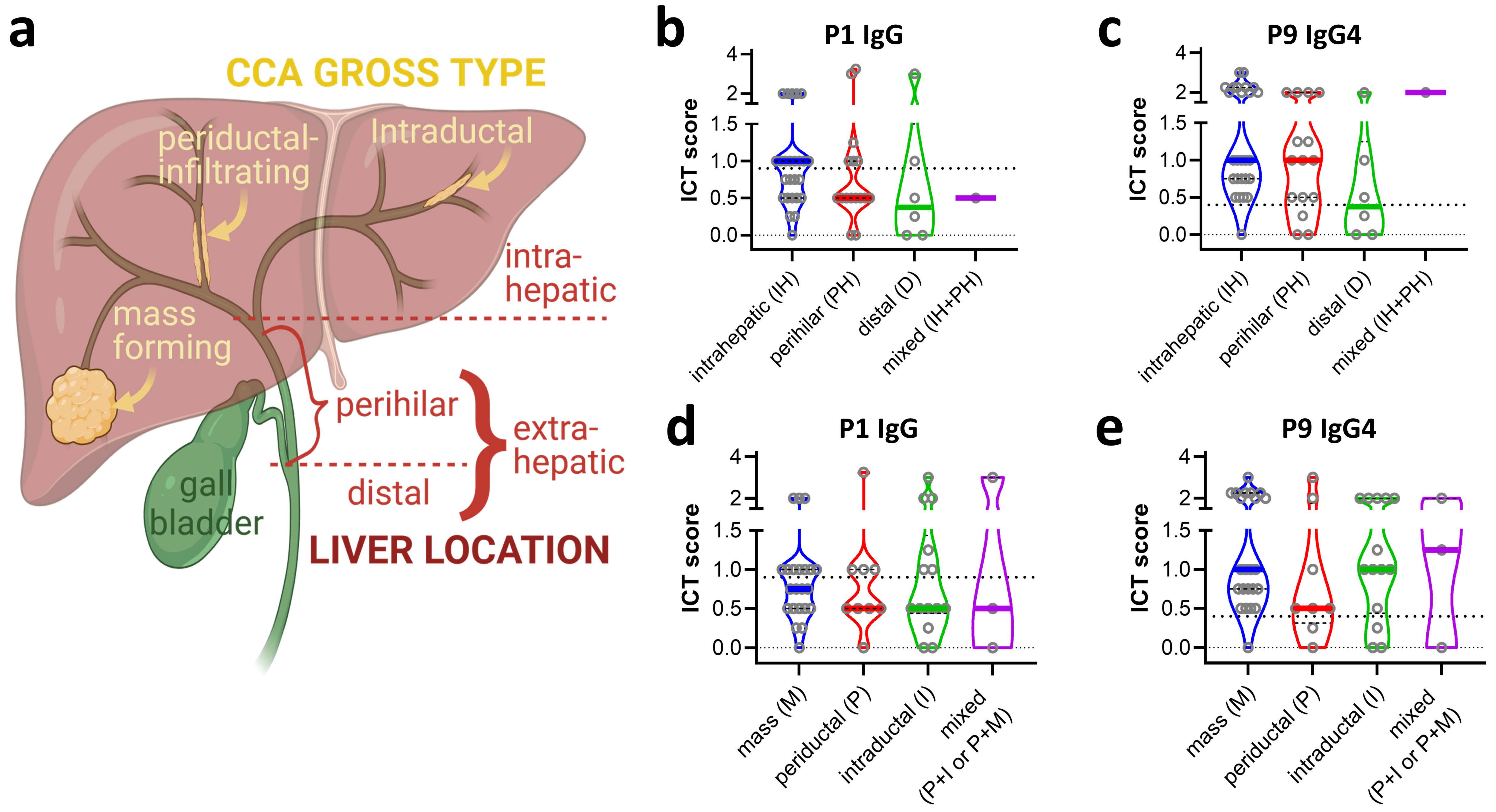

Supplementary Figure 5. ICT score by CCA gross type and location. (a) Liver schematic showing types of CCA marked in yellow, intraductal: cancer cells are contained within the bile duct lumen, periductal infiltrating: the tumors proliferate along the bile duct walls, mass forming: tumor forms a solid mass in the liver parenchyma that has progressed well beyond the confines of the bile duct. Liver location is marked in red: intrahepatic - bile ducts within the liver; perihilar - near the junction of the left and right bile ducts; distal - portion of the common bile duct beyond the gall bladder, closest to the small intestine. CCA liver location plotted against P1 (b) and P9 (c) ICT scores. CCA gross type plotted against P1 (d) and P9 (e) ICT scores. Panels B-E: Violin plots with median as thick colored bar and quartiles marked by dashed lines and individual data points as grey circles. Cutoff for positivity marked with dotted line. Schematic created in BioRender. Smout, M. (2025) *https://BioRender.com/toz528u*

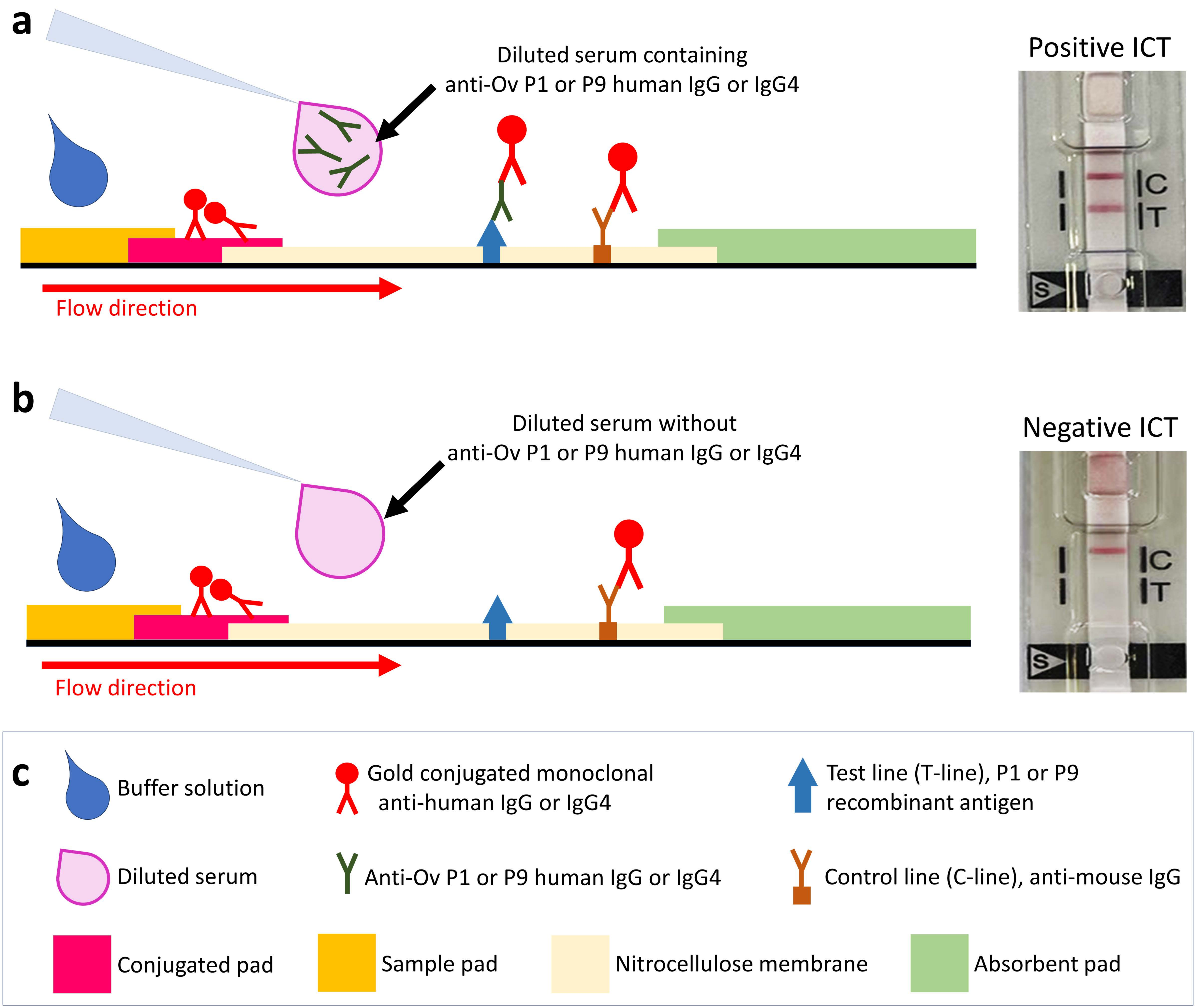

Supplementary Figure 6. Immunochromatographic test (ICT) schematic. Details of how the ICT functions with positive (a) and negative (b) tests shown with schematic key (c) below. Examples of a positive and negative ICTs are shown to the right of each schematic.

Supplementary Table 1. Kappa analyses comparing fecal egg count test (FECT) and immunochromatographic tests for detecting IgG or IgG4 antibodies to O. viverrini excretory/secretory products (ES), recombinant P1 or recombinant P9 antigens.

**Group:**	**OV+**		**OV-**		**Kappa analysis**	
Test	positive	negative	positive	negative	Kappa vs FECT (95% CI)	kappa agreement level
FECT	114	0	0	72	NA	NA
ES IgG4	79	35	1	49	0.565 (0.451-0.680)	moderate
P1 IgG	60	54	12	60	0.325 (0.205-0.444)	fair
P9 IgG4	87	27	9	63	0.610 (0.498-0.722)	substantial
P1+P9	101	13	21	51	0.607 (0.488-0.725)	moderate

Supplementary Table 2. Demographic data for cohorts from which sera were employed to probe the proteome microarray and ICTs.

§ two of 50 CCA cases were FECT positive.

Footnote: EPG, eggs per gram of faces; FECT, fecal egg count technique; ICT, immunochromatographic test; AU, Australia.

**Sample type (analysis method)**	**Countries**	**Areas**	**EPG range**	**Total sample numbers** (female/male)	**Range age (Years)**	**Mean age (Years)**
Healthy subjects (array)	Thailand	Endemic	N/A	50 (39/11)	21 - 60	32.9
Healthy persons USA (array)	USA	Non-endemic	N/A	50 (36/14)	20-59	37
Healthy subjects (ICT)	Thailand	Non-endemic	N/A	15 (15/0)	21 - 87	25.9
Healthy persons AU (ICT)	Australia	Non-endemic	N/A	22 (11/11)	22-58	40.1
Healthy subjects (ICT)	Thailand	Endemic	N/A	37 (26/11)	25 - 58	39.1
Opisthorchiasis, FECT+ve, EPG unknown* (array)	Thailand	Endemic	N/A	50 (16/34)	N/A	N/A
Opisthorchiasis, FECT+ve, EPG known (ICT)	Thailand	Endemic	100 - 5,252	50 (25/25)	26 - 60	49.8
Opisthorchiasis, FECT+ve, EPG known (ICT)	Lao PDR	Endemic	0 - 12,900	64 (40/24)	14 - 63	41.6
Clonorchiasis (array, ICT)	China	Endemic	24 - 39,480	50 (13/37)	15 - 70	42.3
Cholangiocarcinoma (array, ICT)	Thailand	Endemic	2/50§	50 (20/30)	32 - 81	59.7

Supplementary Table 3. Optimal conditions for immunochromatographic test detection.

Rec.Ov/type of antibody detection	Protein	Serum dilution	Antigen concentration (mg/mL)	Running buffer	Cutoff positive
P1 base IgG ICT	OON19686 (cysteine protease)	1:30	2	0.025M Tris base	≥ 1
P1 base IgG4 ICT	OON19686 (cysteine protease)	1:05	2	0.1M Phosphate buffered saline	> 0.5
P5 base IgG ICT	OvM60.2 (mucinase)	1:20	2	0.025M Tris base	≥ 0.5
P5 base IgG4 ICT	OvM60.2 (mucinase)	1:15	2	0.025M Tris base	> 0.5
P6 base IgG ICT	OON17288 (hypothetical)	1:30	2	0.025M Tris base	≥ 0.5
P6 base IgG4 ICT	OON17288 (hypothetical)	1:05	2	0.025M Tris base	≥ 1
P8 base IgG ICT	OON14063 (hypothetical)	1:15	2	0.025M Tris base	≥ 0.5
P8 base IgG4 ICT	OON14063 (hypothetical)	1:05	2	0.025M Tris base	> 0.5
P9 base IgG ICT	OON23642 (isocitrate dehydrogenase)	1:30	2	0.025M Tris base	≥ 0.5
P9 base IgG4 ICT	OON23642 (isocitrate dehydrogenase)	1:10	2	0.025M Tris base	≥ 0.5
