# Supplementary material for: Immunomics-guided biomarker discovery for human liver fluke infection and infection-associated cholangiocarcinoma
Source: Nat Commun. 2025 Jul 1;16:5965. doi: 10.1038/s41467-025-61043-2 (PMC12218120; doi:10.1038/s41467-025-61043-2)
Supplement: Supplementary file 3 — Description of Additional Supplementary Files [file 41467_2025_61043_MOESM3_ESM.pdf]

Description of Additional Supplementary Files

File Name: Supplementary Data 1
Description: List of proteins detected by tandem mass spectrometry from the Opisthorchis viverrini secretome. OvES – O. viverrini soluble excretory/secretory proteins; OvELVs – O. viverrini exosome-like extracellular vesicles; OvMVs - O. viverrini microvesicles.

File Name: Supplementary Data 2
Description: List of Opisthorchis viverrini cDNAs selected for expression in the pXI in vitro translation vector.

File Name: Supplementary Data 3
Description: Diagnostic features of cholangiocarcinoma patients.
