# Supplementary material for: Immunomics-guided biomarker discovery for human liver fluke infection and infection-associated cholangiocarcinoma
Source: Nat Commun. 2025 Jul 1;16:5965. doi: 10.1038/s41467-025-61043-2 (PMC12218120; doi:10.1038/s41467-025-61043-2)
Supplement: Supplementary file 7 — Reporting Summary [file 41467_2025_61043_MOESM7_ESM.pdf]

nature portfolio

Corresponding author(s): Alex Loukas

Last updated by author(s): May 27, 2025

Reporting Summary

Nature Portfolio wishes to improve the reproducibility of the work that we publish. This form provides structure for consistency and transparency in reporting. For further information on Nature Portfolio policies, see our Editorial Policies and the Editorial Policy Checklist.

Statistics

For all statistical analyses, confirm that the following items are present in the figure legend, table legend, main text, or Methods section.

n/a	Confirmed	
☐	☒	The exact sample size (*n*) for each experimental group/condition, given as a discrete number and unit of measurement
☐	☒	A statement on whether measurements were taken from distinct samples or whether the same sample was measured repeatedly
☐	☒	The statistical test(s) used AND whether they are one- or two-sided<br>*Only common tests should be described solely by name; describe more complex techniques in the Methods section.*
☐	☒	A description of all covariates tested
☐	☒	A description of any assumptions or corrections, such as tests of normality and adjustment for multiple comparisons
☐	☒	A full description of the statistical parameters including central tendency (e.g. means) or other basic estimates (e.g. regression coefficient) AND variation (e.g. standard deviation) or associated estimates of uncertainty (e.g. confidence intervals)
☐	☒	For null hypothesis testing, the test statistic (e.g. *F*, *t*, *r*) with confidence intervals, effect sizes, degrees of freedom and *P* value noted<br>*Give P values as exact values whenever suitable.*
☒	☐	For Bayesian analysis, information on the choice of priors and Markov chain Monte Carlo settings
☒	☐	For hierarchical and complex designs, identification of the appropriate level for tests and full reporting of outcomes
☐	☒	Estimates of effect sizes (e.g. Cohen's *d*, Pearson's *r*), indicating how they were calculated

Our web collection on statistics for biologists contains articles on many of the points above.

Software and code

Policy information about availability of computer code

Data collection	NA
Data analysis	Statistical analysis was performed in GraphPad prism 10.4.1

For manuscripts utilizing custom algorithms or software that are central to the research but not yet described in published literature, software must be made available to editors and reviewers. We strongly encourage code deposition in a community repository (e.g. GitHub). See the Nature Portfolio guidelines for submitting code & software for further information.

Data

Policy information about availability of data

All manuscripts must include a data availability statement. This statement should provide the following information, where applicable:

- Accession codes, unique identifiers, or web links for publicly available datasets
- A description of any restrictions on data availability
- For clinical datasets or third party data, please ensure that the statement adheres to our policy

Raw data used in graphs are provided in "Source data" file

Research involving human participants, their data, or biological material

Policy information about studies with human participants or human data. See also policy information about sex, gender (identity/presentation), and sexual orientation and race, ethnicity and racism.

Reporting on sex and gender	Self reported gender used.<br><br>See Supplementary Table 1 for full demographics, summarized below<br><br>Sample type (analysis method)- Total sample numbers (female/male)<br><br>Healthy subjects (array)- 50 (39/11)<br>Healthy persons USA (array)- 50 (36/14)<br>Healthy subjects (ICT) - 15 (15/0)<br>Healthy persons AU (ICT) - 22 (11/11)<br>Healthy subjects (ICT) - 37 (26/11)<br>Opisthorchiasis, FECT+ve, EPG unknown (array) - 50 (16/34)<br>Opisthorchiasis, FECT+ve, EPG known (ICT) - 50 (25/25)<br>Opisthorchiasis, FECT+ve, EPG known (ICT) - 64 (40/24)<br>Clonorchiasis (array, ICT) - 50 (13/37)<br>Cholangiocarcinoma (array, ICT) - 50 (20/30)
Reporting on race, ethnicity, or other socially relevant groupings	Endemic uninfected (Ov-) Thailand samples were from people in North-East Thailand with no detectable eggs in feces and no history of undercooked fish consumption. Non-endemic Thailand samples were from people in Central or Southern Thailand, a region without the parasitic infection. Non-endemic USA or Australia samples were taken from people in these regions with no history of undercooked fish consumption.
Population characteristics	See Supplementary Table 1 for full demographics, summarized below<br><br>Sample type (analysis method) - Countries Areas Age range (Years)<br>Healthy subjects (array) - Thailand Endemic 21 - 60<br>Healthy persons USA (array) - USA Non-endemic 20-59<br>Healthy subjects (ICT) - Thailand Non-endemic 21 - 87<br>Healthy persons AU (ICT) - Australia Non-endemic 22-58<br>Healthy subjects (ICT) - Thailand Endemic 25 - 58<br>Opisthorchiasis, FECT+ve, EPG unknown (array) - Thailand Endemic N/A<br>Opisthorchiasis, FECT+ve, EPG known (ICT) - Thailand Endemic 26 - 60<br>Opisthorchiasis, FECT+ve, EPG known (ICT) - Lao PDR Endemic 14 - 63<br>Clonorchiasis (array, ICT) C- hina Endemic 15 - 70<br>Cholangiocarcinoma (array, ICT) - Thailand Endemic 32 - 81
Recruitment	Opisthorchiasis cases were recruited based on the parasitological confirmation of Opisthorchis viverrini eggs in fecal samples. Similarly, clonorchiasis cases were identified through the detection of Clonorchis sinensis eggs in feces. Cholangiocarcinoma cases from endemic areas were selected following gross and histopathological examinations. Control subjects were included if no parasites were found in their stool examinations and if they had no history of consuming raw fish within the studied population.
Ethics oversight	Khon Kaen University

Note that full information on the approval of the study protocol must also be provided in the manuscript.

Field-specific reporting

Please select the one below that is the best fit for your research. If you are not sure, read the appropriate sections before making your selection.

[x] Life sciences [ ] Behavioural & social sciences [ ] Ecological, evolutionary & environmental sciences

For a reference copy of the document with all sections, see nature.com/documents/nr-reporting-summary-flat.pdf

Life sciences study design

All studies must disclose on these points even when the disclosure is negative.

Sample size	Sample size of 50-114 was determined by available samples from each group.

Data exclusions	No samples were excluded from analysis when sufficient sample was available. Occasionally low sample volume limited the range of possible analyses and these samples are marked in black shown in heatmap figures (Figure 5, Supplementary Figure 3 and 4)
Replication	Additional subject samples were tested (Supplementary Figure 4) and a similar positivity rate was observed (88.6% vs 84.3%).
Randomization	Allocation of infected subjects was not random and was based on age, sex and infection intensity.
Blinding	Blinding was not possible for allocation to defined groups.

Reporting for specific materials, systems and methods

We require information from authors about some types of materials, experimental systems and methods used in many studies. Here, indicate whether each material, system or method listed is relevant to your study. If you are not sure if a list item applies to your research, read the appropriate section before selecting a response.

Materials & experimental systems

n/a	Involved in the study
☐	☒ Antibodies
☒	☐ Eukaryotic cell lines
☒	☐ Palaeontology and archaeology
☒	☐ Animals and other organisms
☐	☒ Clinical data
☒	☐ Dual use research of concern
☒	☐ Plants

Methods

n/a	Involved in the study
☒	☐ ChIP-seq
☒	☐ Flow cytometry
☒	☐ MRI-based neuroimaging

Antibodies

Antibodies used

For the proteome array screening we used the following antibodies and associated reagents:
1. BD Pharmingen 555869 Biotin Mouse Anti-Human IgG1
2. Southern Biotech 9200-08 Mouse Anti-Human IgG4 Fc-BIOT
3. Invitrogen S32357 Streptavidin, Alexa Fluor™ 647 Conjugate.

For the ICT development and probing we used the following reagents and optimisation strategy:
Colloidal gold conjugated mouse monoclonal anti-human IgG (Kestrel BioSciences Co., Pathumthani, Thailand) or IgG4 (Kestrel BioSciences Co.) were sprayed at a conjugate pad and used as the detection probe. Goat anti-mouse IgG (Lampire Biological Laboratories, Pipersville, PA, catalog no. 7455507) was used as the capture antibody at the control line.

Validation

Array optimization:
The reagents listed above have been optimized in-house at UC Irvine and used in dozens of publications by the Felgner group.

ICT optimization and screening.
The ICT test kit has the following components: 1) a nitrocellulose membrane (Sartorius Stedim biotech SA, Goettingen, Germany) onto which 2.0 mg/mL of each recombinant OV antigen is applied as the test (T) line and 1.0 mg/mL of goat anti-mouse IgG (Lampire Biological Laboratories, Pipersville, PA) as the control (C) line by using a XYZ3210 Dispenser (Bio-Dot, Irvine, CA) at a flow rate of 0.1 µL/mm, 2) a conjugate pad (Whatman Schleicher & Schuell, Dassel, Germany) (each colloidal gold-conjugated mouse monoclonal anti-human IgG (Kestrel BioSciences Co.) or IgG4 (Kestrel BioSciences Co.) was sprayed onto a glass microfiber filter (Whatman Schleicher & Schuell) at a flow rate of 1 µL/mm. In addition to these components, a sample pad (Kestrel BioSciences Co.), and an absorbent pad (Kestrel BioSciences Co.) were laminated onto the backing card (Kestrel BioSciences Co.) and the kit finally covered with a plastic housing (Adtec Inc., Oita, Tokyo, Japan). Optimal conditions for immunochromatographic test detection were shown in Supplementary Table 5.

Clinical data

Policy information about clinical studies
All manuscripts should comply with the ICMJEguidelines for publication of clinical research and a completedCONSORT checklist must be included with all submissions.

Clinical trial registration	*Provide the trial registration number from ClinicalTrials.gov or an equivalent agency.*
Study protocol	*Note where the full trial protocol can be accessed OR if not available, explain why.*
Data collection	*Describe the settings and locales of data collection, noting the time periods of recruitment and data collection.*
Outcomes	*Describe how you pre-defined primary and secondary outcome measures and how you assessed these measures.*

Plants

Seed stocks

Report on the source of all seed stocks or other plant material used. If applicable, state the seed stock centre and catalogue number. If plant specimens were collected from the field, describe the collection location, date and sampling procedures.

Novel plant genotypes

Describe the methods by which all novel plant genotypes were produced. This includes those generated by transgenic approaches, gene editing, chemical/radiation-based mutagenesis and hybridization. For transgenic lines, describe the transformation method, the number of independent lines analyzed and the generation upon which experiments were performed. For gene-edited lines, describe the editor used, the endogenous sequence targeted for editing, the targeting guide RNA sequence (if applicable) and how the editor was applied.

Authentication

Describe any authentication procedures for each seed stock used or novel genotype generated. Describe any experiments used to assess the effect of a mutation and, where applicable, how potential secondary effects (e.g. second site T-DNA insertions, mosiacism, off-target gene editing) were examined.
